# Growth Kinetics and Transmission Potential of Existing and Emerging Field Strains of Infectious Laryngotracheitis Virus

**DOI:** 10.1371/journal.pone.0120282

**Published:** 2015-03-18

**Authors:** Sang-Won Lee, Carol A. Hartley, Mauricio J. C. Coppo, Paola K. Vaz, Alistair R. Legione, José A. Quinteros, Amir H. Noormohammadi, Phillip F. Markham, Glenn F. Browning, Joanne M. Devlin

**Affiliations:** 1 Asia-Pacific Centre for Animal Health, Faculty of Veterinary and Agricultural Sciences, The University of Melbourne, Victoria, 3010, Australia; 2 Asia-Pacific Centre for Animal Health, Faculty of Veterinary and Agricultural Sciences, The University of Melbourne, Werribee, 3030, Australia; University of Liverpool, UNITED KINGDOM

## Abstract

Attenuated live infectious laryngotracheitis virus (ILTV) vaccines are widely used in the poultry industry to control outbreaks of disease. Natural recombination between commercial ILTV vaccines has resulted in virulent recombinant viruses that cause severe disease, and that have now emerged as the dominant field strains in important poultry producing regions in Australia. Genotype analysis using PCR—restriction fragment length polymorphism has shown one recombinant virus (class 9) has largely replaced the previously dominant class 2 field strain. To examine potential reasons for this displacement we compared the growth kinetics and transmission potential of class 2 and class 9 viruses. The class 9 ILTV grew to higher titres in cell culture and embryonated eggs, but no differences were observed in entry kinetics or egress into the allantoic fluid from the chorioallantoic membrane. *In vivo* studies showed that birds inoculated with class 9 ILTV had more severe tracheal pathology and greater weight loss than those inoculated with the class 2 virus. Consistent with the predominance of class 9 field strains, birds inoculated with 10^2^ or 10^3^ plaque forming units of class 9 ILTV consistently transmitted virus to in-contact birds, whereas this could only be seen in birds inoculated with 10^4^ PFU of the class 2 virus. Taken together, the improved growth kinetics and transmission potential of the class 9 virus is consistent with improved fitness of the recombinant virus over the previously dominant field strain.

## Introduction

Infectious laryngotracheitis virus (ILTV) causes acute respiratory disease in chickens, resulting in significant economic losses worldwide. Outbreaks of infectious laryngotracheitis (ILT) are controlled using attenuated live vaccines produced by serial passages in embryonated hen eggs or tissue cultures, and these vaccines are in widespread use in the poultry industry [1]. More recently, recombinant viral vector vaccines that express ILTV proteins have been developed and these ‘new generation’ vaccines are in current use in the USA and elsewhere. While these vaccines avoid the issue of residual virulence, they appear to be less effective than conventionally attenuated vaccines in inducing an adequate immune response to completely protect chickens from ILTV infection [2,3].

Since late 2007 a large number of outbreaks of ILT have been seen in commercial poultry in Australia in both Victoria and New South Wales. Genotype analysis using PCR—restriction fragment length polymorphism analysis (PCR-RFLP) showed that the majority of these outbreaks were caused by infection with two previously unknown genotypes of ILTV, class 8 and class 9 [4,5]. The emergence of the class 8 and 9 genotypes occurred as a result of the concurrent use of both the Australian origin SA2/A20 and European origin Serva vaccine strains, which resulted in multiple natural recombination events and the creation of these two classes of virulent recombinant viruses [6]. The class 9 recombinant virus is now the dominant field strain in some important poultry producing regions of Australia, and has largely replaced the previously dominant class 2 genotype of ILTV [4].

The reasons for the current predominance of the class 9 virus and the displacement of the class 2 genotype are not known. The aim of this study was to investigate whether the displacement of the class 2 viruses can be explained by the increased fitness of the class 9 virus and to characterize the transmission potential of a class 9 field strain of ILTV. These studies included a comparative *in vitro* analysis of the growth properties of class 2 and class 9 viruses, as well as an examination of their transmission potential *in vivo*.

## Materials and Methods

### Viruses

The class 2 genotype virus used in this study was isolate V1–99 [5,7], while the class 9 isolate was first described by Blacker et al. [4] and was later sequenced by Lee et al. [6]. Viruses were amplified by 6 passages on chorioallantoic membranes (CAM) of 10 day old specific pathogen free (SPF) embryonated hen eggs, followed by 3 to 4 passages on the chicken liver tumour cell line LMH [8].

### Cell culture

LMH cell monolayers were cultured as described previously [9]. Virus titres in plaque forming units per mL (PFU/mL), growth kinetics and cell-to-cell spread were measured essentially as described by Devlin et al (2006b), although the area, rather than the diameter, of 19 to 26 individual plaques was measured, using Image J [10]. Assays to compare the kinetics of virus attachment to and entry into LMH cells and the egress of virus from the CAM into the allantoic fluid were performed and analysed as described [11].

### Challenge and transmission study

The study protocol was approved by the University of Melbourne’s Animal Ethics Committee (Permit number AEC 1312726.1) which follows the Australian code for the care and use of animals for scientific purposes [12]. This permit approved the euthanasia of birds using halothane inhalant. Seven groups of 10 twenty-eight-day-old commercial broiler birds, including one negative control (mock inoculated) group, were used to investigate the consequences and transmission of infection with three different doses of class 2 and class 9 field strains of ILTV. Birds were weighed and assigned randomly to each group, with each group housed in a separate isolator and provided with irradiated food and water *ad libitum*. Birds were inoculated with 10^2^, 10^3^ or 10^4^ PFU of either class 2 or class 9 ILTV by eye drop and intra-tracheal instillation of inoculum, with half of the dose administered to each site. The mock-inoculated group received an equivalent volume of sterile Dulbecco’s Minimal Essential Medium (DMEM) cell culture medium. At the conclusion of inoculating all the birds in each group 10 age-matched naïve birds were transferred into each isolator as ‘in-contact’ birds. All birds were weighed and conjunctival and tracheal swabs were collected at multiple time points post-infection. All in-contact birds were euthanased at 8 days post-contact. Blood samples were collected from directly inoculated birds at 21 days post infection (dpi) immediately before they were euthanased, and serums tested for ILTV antibody using the TropELISA-ILT kit (TropBio). Post mortem analyses included weighing birds and collection of tracheal tissue for histopathology.

### Quantitation of ILTV on swabs

Swabs were collected into 1 mL of viral transport medium (DMEM, 3% v/v foetal bovine serum and 100 μg ampicillin/mL) and DNA extracted from 200 μL of the sample using the Vx reagent and consumable packs on the QIAextractor automated system (Qiagen). The concentration of ILTV genome in each sample was determined using a quantitative PCR (qPCR) assay targeting the UL15 gene of ILTV, incorporating a standard curve to enable estimation of genome copy numbers in the sample [13].

### Histopathology

Transverse sections of the proximal trachea were collected into Bouin’s fixative prior to histopathological examination. After staining with haematoxylin and eosin, sections were examined by light microscopy and scored by an operator blinded to groups using a previously described system [14]. The scoring system is based on the observation of inflammatory infiltration, the integrity of the epithelium and the presence of syncytia and intranuclear inclusion bodies. Lesion scores were compared between groups using a Mann-Whitney U test.

## Results

### In vitro replication and growth kinetics

**Fig 1:**
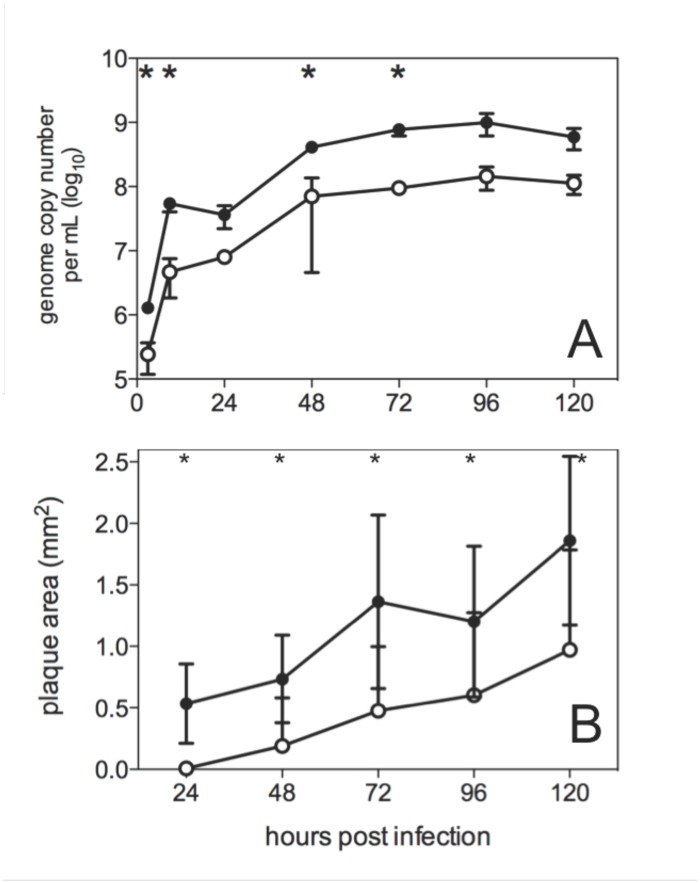
Kinetics of virus growth (A) and plaque development (B) of class 2 (open circles) and class 9 (closed circles) ILTV in LMH cells. (A) LMH cells were inoculated at multiplicity of infection of 0.001 and inocula removed at one hour post-inoculation. Triplicate wells were harvested at 24 hour intervals after infection and virus genome concentrations in the cell-free supernatant determined using qPCR. Error bars indicate standard deviations and asterisks indicate time points at which the mean concentrations of the two viruses were significantly different (P<0.05; Student’s t-test). (B) LMH cells were inoculated with virus, after which they were overlaid with methyl cellulose medium and incubated at 37°C. Nineteen to 26 individual plaques were photographed at each time point, and plaque area measured using Image J, calibrated using a stage micrometer. Plaques induced by each of the viruses differed significantly in mean area at each time point (P < 0.004; Student’s t-test).

**Fig 2:**
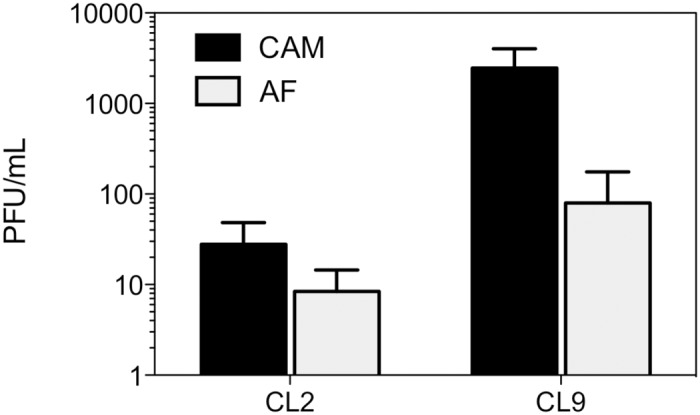
Comparison of titres of class 2 and class 9 ILTV in CAM extracts and allantoic fluid. Virus was inoculated onto the CAMs of embryonated hen eggs (5 x 10^3^ PFU for each virus), which were then incubated for 6 days. Mean virus titres are shown. Error bars indicate standard deviations.

**Fig 3:**
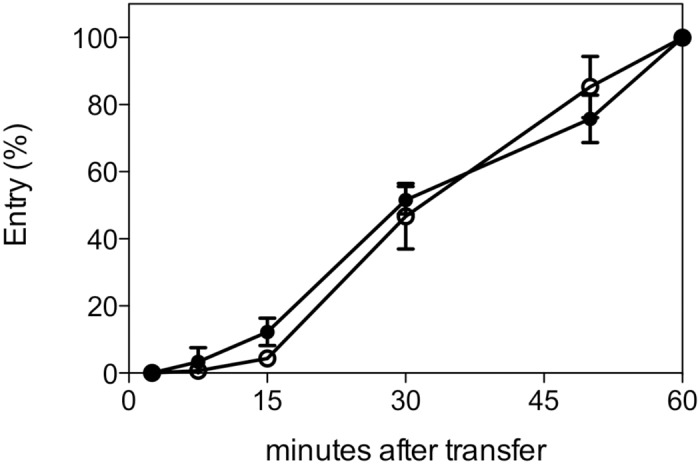
Entry kinetics of class 2 (open circles) and class 9 (closed circles) ILTV into LMH cells. Entry at different time points was calculated by comparing the number of plaques formed after inoculation for that period of time to the number of plaques formed after an inoculation period of 60 minutes. The means and standard deviations (error bars) from 3 independent experiments are shown.

Viral replication studies performed in LMH cells (Fig. 1) showed that both viruses approached peak levels by 48 hours post-infection, with the class 9 virus reaching significantly higher levels than the class 2 virus at 4 of the 7 time points examined (P values 0.013, 0.017, 0.012 and 0.016 for 3, 9, 48 and 72 hours post infection, respectively). Consistent with this finding, the class 9 virus generated significantly larger plaques than the class 2 virus at every time point (Fig. 1, P values ≤0.02), suggesting that the class 9 virus spread more efficiently from cell to cell than the class 2 virus. The class 9 virus also reached significantly higher levels in CAMs than the class 2 virus (Fig. 2, P = 0.02). However, the class 9 virus did not show faster entry kinetics into LMH cells (Fig. 3) nor improved efficiency of egress into the allantoic fluid after CAM inoculation (Fig. 2). In fact, while the class 9 virus reached significantly higher levels than the class 2 virus in the CAM, a comparison of the relative proportions of virus in the CAM and allantoic fluid indicated that most of the class 9 virus was retained in the CAM, with significantly lower proportions released into the allantoic fluid than was seen with the class 2 virus (P = 0.02). Taken together these data showed that the class 9 virus had improved *in vitro* replication kinetics in LMH cells and in CAMs compared to the class 2 virus.

### In vivo replication and pathogenesis

Commercial broilers were inoculated with either the class 2 or class 9 virus at 3 different doses (10^2^, 10^3^, 10^4^ PFU/bird) and 10 age-matched birds were added to each group after inoculation in order to compare bird-to-bird transmission of these two viruses. By day 5 post-infection, all birds directly inoculated with the highest dose of class 9 virus had to be euthanased because of the severity of their clinical signs. Groups that had been directly inoculated with 10^2^ or 10^3^ PFU of class 9 virus, or 10^3^ PFU of the class 2 virus, had significantly (P = 0.001, 0.006 or 0.003 respectively) lower mean weight gains than that of the uninfected control group (Table 1). The mean weight gains of the groups of in-contact birds were similar to those of the directly inoculated birds. However given that males increase weight more rapidly than females (and the problems associated with accurate sexing of broilers by sight at the age the birds were allocated to groups) the variation in the proportions of males and females in groups receiving 10^3^ PFU of virus may have affected the ability to detect differences between birds infected with class 2 or class 9 viruses.

**Table 1:** Mean percentage weight gain (± standard deviation) of birds after direct inoculation with class 2 or class 9 ILTV, or after contact with inoculated birds.

Inoculum	Inoculated (6 dpi)	Sex (F/M)	In-contact (8 dpi)	Sex (F/M)
**Uninfected**	63.75 ± 16.50^a^	5/5	85.95 ± 13.77^a^	5/5
***Class 2 ILTV***				
**10^2^ PFU**	65.70 ± 24.79^a^	4/5	86.14 ± 6.81^a^	7/3
**10^3^ PFU**	48.08 ± 9.78^b^	4/6	59.72 ± 14.02^b^	8/2
**10^4^ PFU**	56.75 ± 9.58^a,c^	3/6	79.97 ± 10.47^a^	6/4
***Class 9 ILTV***				
**10^2^ PFU**	36.60 ± 15.60^b^	4/4	62.16 ± 9.50^b^	6/4
**10^3^ PFU**	49.73 ± 12.73^b,c^	3/5	80.11 ± 6.47^a^	2/4
**10^4^ PFU**	NA*		52.48 ± 12.85^b^	6/4

Values with identical superscripts in the same column were not significantly different (Student’s t-test; P ≥ 0.05)

* NA = not available

Surviving birds that were directly inoculated with the highest dose of the class 9 virus had significantly (P = 0.004 and 0.001 for 10^2^ and 10^3^ PFU groups respectively) more severe tracheal histopathology scores than those inoculated with the class 2 virus, even at 21 dpi (Table 2). There was a more distinct difference (P ≤ 0.001) between the groups of in-contact birds (Table 2), probably because the samples were taken at a time point closer to the most acute phase of infection. In-contact infection with the class 9 virus resulted in more severe tracheal histopathology scores than in-contact infection with the class 2 viruses regardless of the dose.

**Table 2:** Median (range) tracheal histopathology scores of birds after direct inoculation with class 2 or class 9 ILTV, or after contact with infected birds.

Inoculum	Inoculated (21 dpi)	In-contact (8 dpi)
**Uninfected**	2 (1–2)^a^	1 (0–2)^a^
***Class 2 ILTV***		
**10^2^ PFU**	2 (1–3)^a,b^	1 (0–2)^a^
**10^3^ PFU**	2 (1–3)^a,b^	0.5 (0–3)^a^
**10^4^ PFU**	3 (1–3)^b,c^	1 (0–3)^a^
***Class 9 ILTV***		
**10^2^ PFU**	3 (2–4)^c,d^	4 (4–5)^b^
**10^3^ PFU**	3 (2–5)^d^	4 (3–5)^b^
**10^4^ PFU**	NA*	4 (3–5)^b^

Values with identical superscripts in each column were not significantly different (Mann-Whitney U test; P ≥ 0.05).

* NA = not available

**Fig 4:**
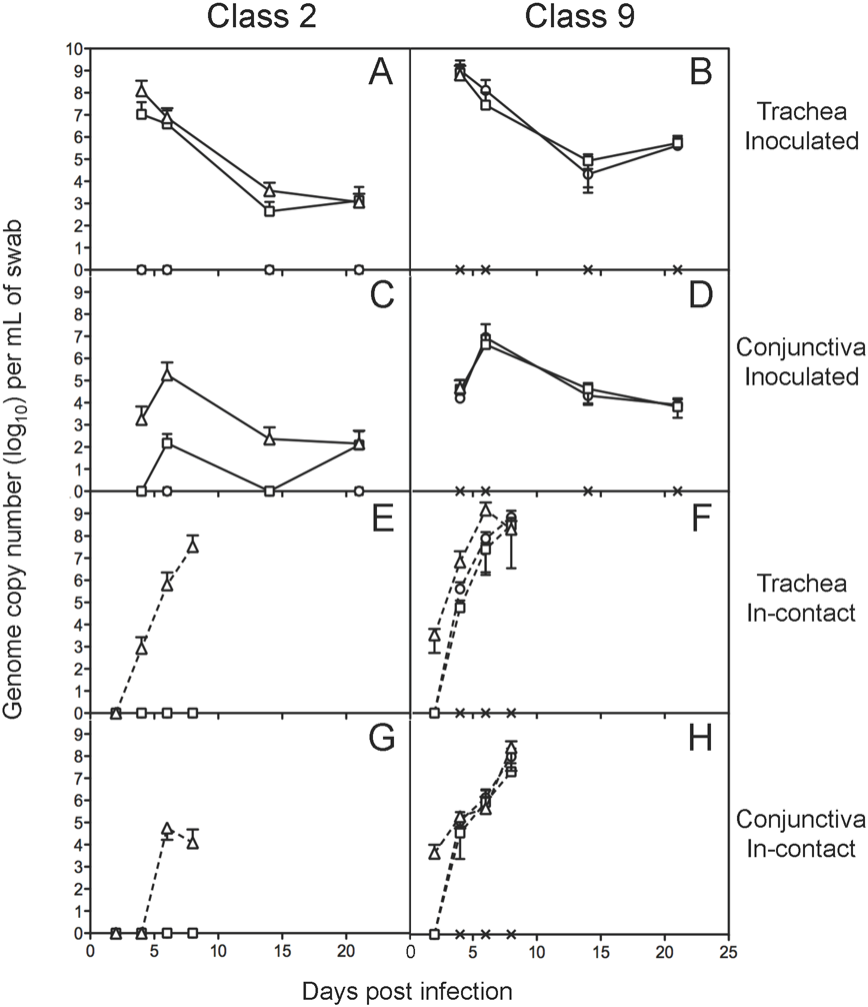
Virus shedding in class 2 ILTV (left hand panels) and class 9 ILTV (right hand panels) infected birds as detected by tracheal (A, B, E, F) and conjunctival (C, D, G, H) swabbing. Swabs were collected from directly inoculated (A-D, solid line) or in-contact (E-H, broken line) birds inoculated with 10^2^ (circles), 10^3^ (squares) or 10^4^ (triangles) PFU of virus or sterile media (x). Genome concentrations were determined using an ILTV-UL15-specific qPCR. Mean log_10_ viral genome concentrations for each group are shown, with error bars indicating standard deviations.

Virus shedding in birds inoculated with the class 2 virus was dose-dependent (Fig. 4). Birds inoculated with 10^2^ PFU did not develop a detectable productive infection, and only in the birds inoculated with the highest dose was virus detectable in every swab at every time point. Inoculation with the class 9 virus resulted in high levels of infection in birds regardless of dose, and virus was detectable at all doses at every time point tested post-infection. Consistent with these findings, only birds inoculated with the highest dose of the class 2 virus and 100 and 1,000 PFU of the class 9 virus seroconverted to ILTV during the 21 day infection (S1 Fig.). The class 9 virus was also transmitted to the in-contact birds at every dose, in contrast to the class 2 virus, which was only transmitted to in-contact birds from those birds inoculated with the highest dose. The greatest difference in the levels of virus shed between the two classes was observed in the conjunctiva and might suggest that conjunctival, rather than tracheal, shedding is a more efficient method of transmission to in contact birds. Clinical signs were not specifically measured in this study but, similar to our previously performed experimental inoculations [15,16], the observed clinical signs of disease in inoculated birds included dyspnea, mild conjunctivitis and a depressed demeanour. Coughing was not observed.

## Discussion

In Australia, as in some other parts of the world, there have been an increasing number of ILT outbreaks since 2007. Natural recombination events between distinct vaccine strains have led to the emergence of recombinant class 8 and 9 genotype viruses, with the class 9 virus now appearing to have displaced the previously dominant class 2 genotype ILTV in some poultry producing areas. The study described here suggests the shift towards the class 9 virus may have occurred because of the improved fitness of the class 9 virus over class 2 viruses. This increased fitness is indicated by the more efficient replication kinetics of the class 9 virus, its infectivity at lower doses and its enhanced transmissibility to in-contact birds.

The attenuation of the parental vaccine viruses of the class 9 recombinant by multiple passages *in vitro* is likely to have selected for viruses well adapted to growth *in vitro*, and this is consistent with the properties demonstrated by the class 9 virus in this study. Class 2 and class 9 ILTVs share 99.6% pairwise nucleotide identity [7], and have 142 non-synonymous differences, within 49 unique ORFs. However the genome of the virulent class 9 virus is derived from 2 attenuated vaccine strains [6], and therefore the same SNPs do not confer virulence in these vaccine/parent viruses. This suggests that a constellation of genetic differences, rather than any single nucleotide polymorphism, may be responsible for the increased virulence. Differences between class 2 and class 9 genotypes are detected in genes that have been directly identified as ILTV virulence genes, including glycoprotein G [15,17] and UL0 [18]. Differences also occur in genes that have been indirectly associated with virulence (by comparing the genomes of vaccine and field strains), including ORF B [19], ORF C, ORF F, UL17, UL23, UL27, UL36, UL39 and US5 [20]. The relevance of these ORFs/SNPs to virulence has not been directly confirmed by mutagenesis or gene deletion in the original studies and there is sufficient variation in these ORFs across different ILTV strains to obscure any specific link. To further complicate these associations, some vaccine strains can be as virulent as field strains when administered by similar routes, and at similar doses [16]. Genotype analysis and mutagenesis combined with direct comparisons of virulence are necessary to better define the genotypic determinants of virulence in ILTV.

The class 9 virus had significantly better growth kinetics *in vitro*, reflected in its capacity to grow to higher levels and to spread more rapidly from cell to cell. The identical entry kinetics (Fig. 3) of both viruses suggests the growth advantage of class 9 ILTV is at later replication stages. Upon inoculation of the conjunctival or tracheal mucosa, ILTV attaches and enter mucosal epithelial cells [21]. ILTV infection of mucosal epithelial explant cultures from the trachea and conjunctiva has demonstrated local extension across the epithelial cells, resulting in plaque-like sites of infection that increase in both in latitude and depth until a proportion of plaques extend beyond the basement membrane (BM) [22]. It has been suggested that penetration of the BM by ILTV is not as efficient as in other alphaherpesviruses (such as bovine herpesvirus 1, suid herpesvirus 1 and herpes simplex virus 1), and that this is consistent with the lack of viraemia being detected in ILTV-infected birds. Our current study suggests that the improved growth kinetics of the class 9 virus may ultimately result in more efficient spread through the BM.

The improved growth and transmission of class 9 ILTV demonstrated here is consistent with the displacement of class 2 ILTV by the class 9 virus in the field. It appears that this recombinant ILTV has been selected in the field towards a more virulent phenotype. The evolution of the avian alphaherpesvirus Marek’s disease virus (MDV) towards increasing virulence has been described in detail previously [23,24]. Both ILTV and MDV vaccines provide imperfect protection, reducing clinical signs, but not preventing virus shedding or transmission to susceptible hosts [1,25]. Comparison of unvaccinated and vaccinated populations has suggested that imperfect MDV vaccines may drive evolution of virulence by reducing the severity of clinical signs, and thus ensuring longer survival of infected hosts and extending the length of the infectious period in birds infected with virulent MDV strains [26,27]. Although there are a number of important differences in the pathogenesis of MDV and ILTV, including the more acute ILTV-related mortality that will significantly shorten the infectious period of infected birds, vaccination has been associated with virulence evolution in several different host-pathogen systems [28]. While the detail of the pressures that drive selection for virulence in ILTV remain to be determined, the generation of ILTV vaccines that are both unable to revert to virulence and are capable of providing sterilizing immunity would seem to offer the ideal scenario to provide control of this important poultry disease.

## Supporting Information

S1 Fig Antibody levels of directly inoculated birds at day 0 and day 21 post infection with class 2 (dashed line) and class 9 viruses (solid line). ELISA titres were determined in serums from uninfected birds (x) or birds infected with 10^2^ (circles), 10^3^ (squares) or 10^4^ (triangles) PFU of virus.
(TIFF)
