# Supplementary figures and images for: Growth Kinetics and Transmission Potential of Existing and Emerging Field Strains of Infectious Laryngotracheitis Virus
Source: PLoS One. 2015 Mar 18;10(3):e0120282. doi: 10.1371/journal.pone.0120282 (PMC4365042; doi:10.1371/journal.pone.0120282)

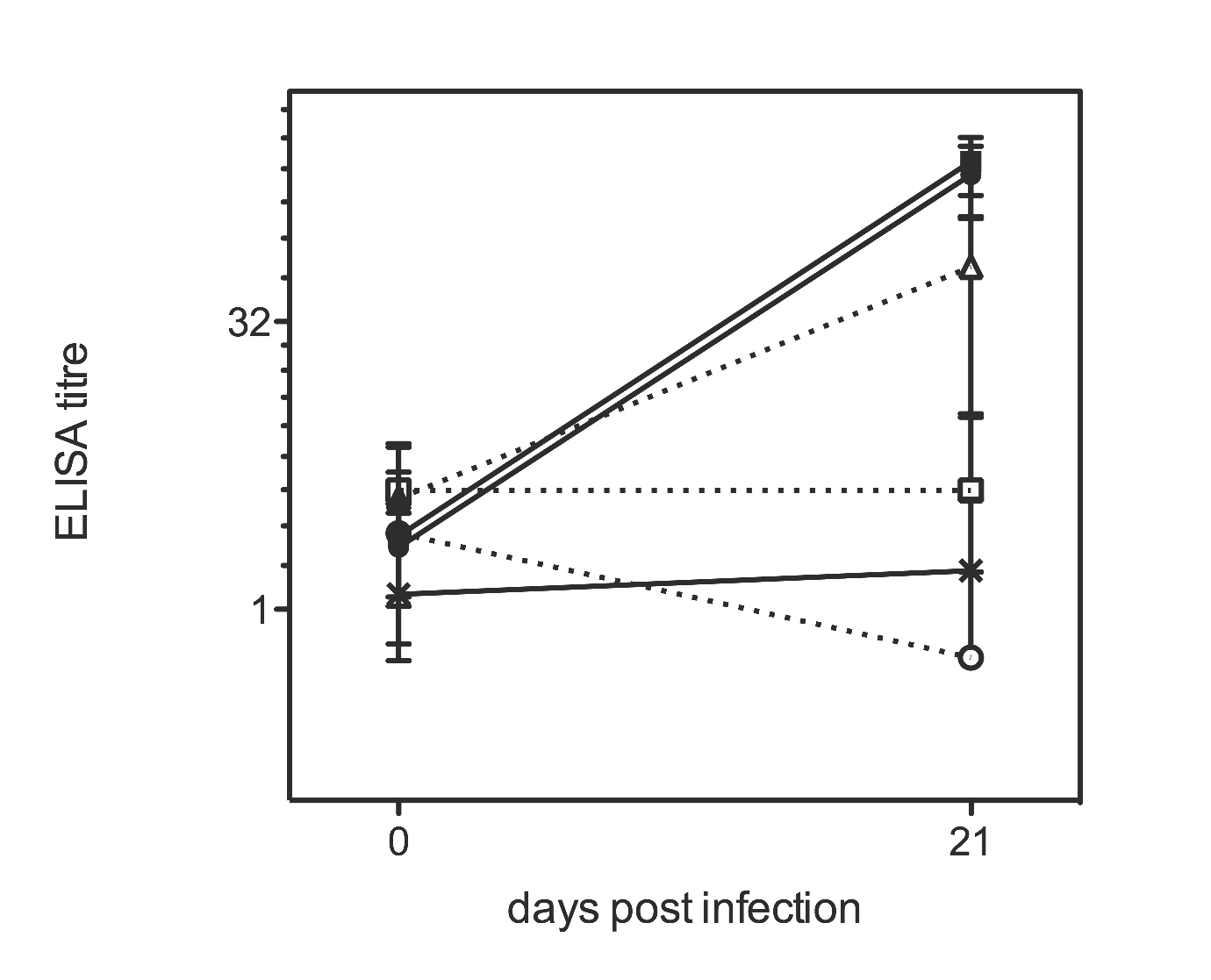

Supplement: S1 Fig — ELISA titres were determined in serums from uninfected birds (x) or birds infected with 102 (circles), 103 (squares) or 104 (triangles) PFU of virus. (TIFF) [file pone.0120282.s001.tiff]
